# Investigations into the Antifungal, Photocatalytic, and Physicochemical Properties of Sol-Gel-Produced Tin Dioxide Nanoparticles

**DOI:** 10.3390/molecules27196750

**Published:** 2022-10-10

**Authors:** Sirajul Haq, Nadia Shahzad, Muhammad Imran Shahzad, Khaled Elmnasri, Manel Ben Ali, Alaa Baazeem, Amor Hedfi, Rimsha Ehsan

**Affiliations:** 1 Department of Chemistry, University of Azad Jammu and Kashmir, Muazaffabad 13100, Pakistan; 2 US-Pakistan Centre for Advance Studies in Energy, National University of Science and Technology (NUST), Islamabad 44000, Pakistan; 3 Nanoscience and Nanotechnology Department, National Centre for Physics (NCP), Islamabad 44000, Pakistan; 4 Higher institute of Biotechnology, University of Manouba, ISBST, BVBGR-LR11ES31, Biotechpole Sidi Thabet, Ariana 2010, Tunisia; 5 Department of Biology, College of Science, Taif University, P.O. Box 11099, Taif 21944, Saudi Arabia

**Keywords:** antifungal activity, tin dioxide, sol-gel, tetragonal, photocatalysis, solar-light

## Abstract

Transmission electron microscopy (TEM), atomic force microscopy (AFM), X-ray diffraction (XRD), energy dispersive X-ray (EDX), scanning electron microscopy (SEM), diffuse reflectance spectroscopy (DRS), and Fourier transform infrared (FTIR) spectroscopy were applied to evaluate the tin dioxide nanoparticles (SnO_2_ NPs) amalgamated by the sol-gel process. XRD was used to examine the tetragonal-shaped crystallite with an average size of 26.95 (±1) nm, whereas the average particle size estimated from the TEM micrograph is 20.59 (±2) nm. A dose-dependent antifun3al activity was performed against two fungal species, and the activity was observed to be increased with an increase in the concentration of SnO_2_ NPs. The photocatalytic activity of SnO_2_ NPs in aqueous media was tested using Rhodamine 6G (Rh-6G) under solar light illumination. The Rh-6G was degraded at a rate of 0.96 × 10^−2^ min for a total of 94.18 percent in 350 min.

## 1. Introduction

The fungi are the heterotrophic eukaryotes that are unable to make their own food. These multicellular eukaryotes are ubiquitous, thus fungal infections are common throughout the world. In humans, fungal infections are mostly caused when a fungus attacks over the low immunity area of the body that is adaptive to it. Fungi can live in plants, soil, air, water and in human body naturally [1]. Like other microbial organisms, some fungi are harmful, while some are useful. When a harmful fungus attacks the human body the victim complains of itching, swelling and redness depending on the attacked area of the body. Fungi cause both surface and systemic infections and can have lethal outcomes if diagnosed at the later stages [2]. 

The attack of pathogens, especially fungi, has put the food security at risk and, according to a rough estimate, almost one-third of annual crops are lost due to the attack and invasion of these harmful pathogens [3]. Economically valuable crops are harmed by pathogenic fungi at pre-harvest or post-harvest stages. The fungicides used for their control are imparting a damaging effect on both humans and the environment. Thus, silver nanoparticles have been reported for the control of phytopathogenic fungi as these NPs cause growth restriction of such fungi without disturbing the environment [4]. Solvo-thermally synthesized gold NPs have also been advertised for antifungal activity against the candida species [5]. The ZnO NPs synthesized by biological methods using extracts like *Allium cepa*, garlic, parsley, *Dolichos lablab* L. and *Sphingomonas paucimobilis* have also been reported in the literature showing affective fungal growth inhibition, mostly of candida species [6]. 

The reduction of organic dyes from water reservoirs are a major concern in this industrial world and the damages related to the presence of organic substances in aquatic environments is unmeasurable. Rhodamine 6G is a heterocyclic cationic polar dye belonging to the Xanthene family and has strong absorption in the visible region [7]. Rhodamine 6G in used in the field of hydraulics as fluorescent tracer to visualize flow patterns and also is commonly used also as a sensitizer. The discharge of rhodamine 6G into the aqueous medium is harmful for humans and longer term exposure results in multiple health issues such as vomiting, increase in heart rate, lung cancer, skin cancer and, in some cases, delay in physiological development. Therefore, it is highly necessary to degrade organic substances from waste water before they accumulate in the environment causing irreversible damage [8]. Photocatalysis involves the production of the hydroxyl radical and superoxide anion, which are generated by absorption of radiation by the catalyst. SnO_2_ is widely used due to its nontoxic effect, stability and strong oxidizing properties [9].

SnO_2_ is one of the best semiconductors, having shape dependent properties and a band gap of 3.6 eV [10]. The SnO_2_ NPs have potential to degrade organic dye and help in the protection of the environment [11]. At the nano-scale, SnO_2_ exhibits exceptional properties owing to its high surface area to volume ratio, which makes it a unique photocatalyst [12]. The nano-sized SnO_2_ is an efficient catalyst for oxidation of organic compounds due to the presence of a high number of surface active groups [13]. The large surface area of SnO_2_ NPs having a size below 10 nm has more reaction sites, which increases the photocatalytic efficacy, which might also be attributed to the large electron-hole pair separation [14]. The Sol-gel synthesis of SnO_2_ NPs is preferred over other methods, because it is easy to handle, provides better control over the particle size and is economic [15].

The current research concerns the sol-gel synthesis of SnO_2_ NPs for antifungal activity and photodegradation of rhodamine 6G. The as-manufactured SnO_2_ NPs were characterized by manipulating SEM, XRD, EDX, AFM, TEM, DRS and FTIR spectroscopy. The antifungal activity was performed against the selected fungus species using the Agar well diffusion method. The selection of fungi is purely based on the availability of the fungal strain and its toxicity. The degradation of rhodamine 6G was brought under solar light irradiation and the reaction parameters were determined by a set of mathematical equations.

## 2. Results

### 2.1. XRD Analysis 

**Figure 1:**
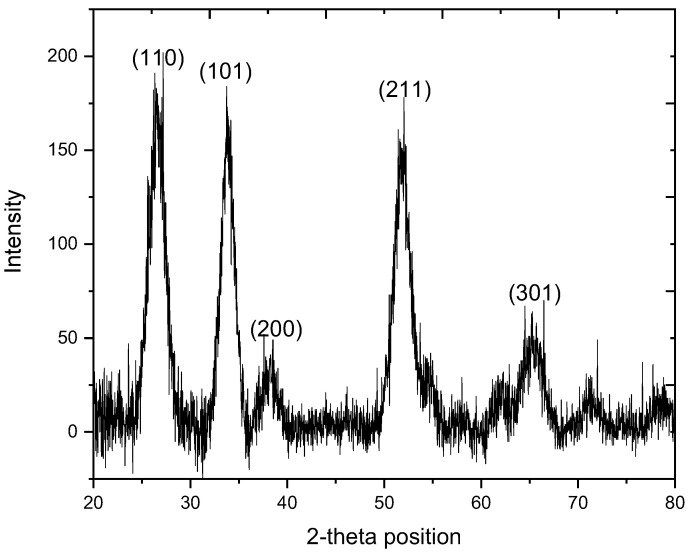
XRD pattern of SnO_2_ NPs.

The XRD pattern of SnO_2_ NPs exhibited in Figure 1 shows the characteristic peaks along with corresponding hkl values for SnO_2_ at 2θ 26.34 (110), 33.68 (101), 38.06 (200), 52.00 (211) and 65.21 (301), which harmonized with the diffraction bands listed in the JCPDS card no. 01-077-0449 assigned to the cubic geometry of crystals. The noisy XRD pattern with broad diffraction band suggests the presence of both amorphous and crystalline phase in the sample [16]. The sharpness of diffraction bands suggest that some portion of the synthesized material is highly crystalline while the varied width and intensity shows a wide range distribution of crystallite size. The average crystallite size for SnO_2_ NPs enumerated by Debye-Scherrer equation is 26.95 nm with 0.39% imperfection, found in the crystal.

### 2.2. EDX Analysis 

**Figure 2:**
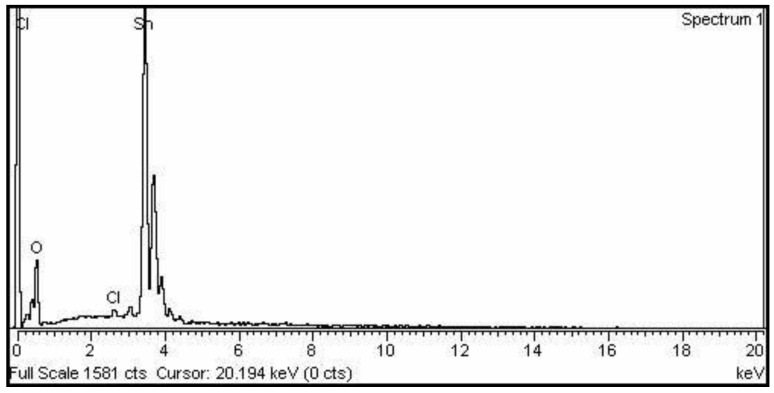
EDX pattern of SnO_2_ NPs.

In the EDX spectrum of SnO_2_ NPs (Figure 2), O is responsible for the peak at 0.3 keV, while Sn is responsible for a series of sharp bands in the 3.5–4 keV range, as well as a very tiny signal at 2.6 due to the presence of Cl. According to the EDX analysis, the synthesized SnO_2_ NPs have a stoichiometric composition of Sn and O, with a trace of Cl as an impurity. According to EDX statistics, Sn, O, and Cl have weight percentages of 78.7, 20.2 and 1.1 percent, respectively.

### 2.3. SEM Analysis 

**Figure 3:**
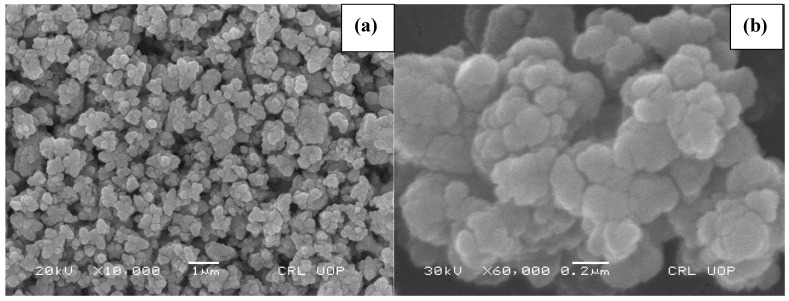
SEM micrographs of SnO_2_ NPs. (**a**) ×10,000; (**b**) ×60,000.

The structural analysis of SnO_2_ NPs was carried out via SEM as shown in the low and high magnified micrographs (Figure 3a,b). The images reveal that flat shaped particles of different size are formed by the aggregation of small particles. Each flat shaped particle constitutes 2 to 9 small particles depending upon the size, and the cracks observed in the flat shaped particles are actually the boundaries of the aggregated particles. The size of the flat shaped particles predicted from SEM micrographs range from 78 to 114 nm with an average size of 98.56 nm.

### 2.4. TEM Analysis 

**Figure 4:**
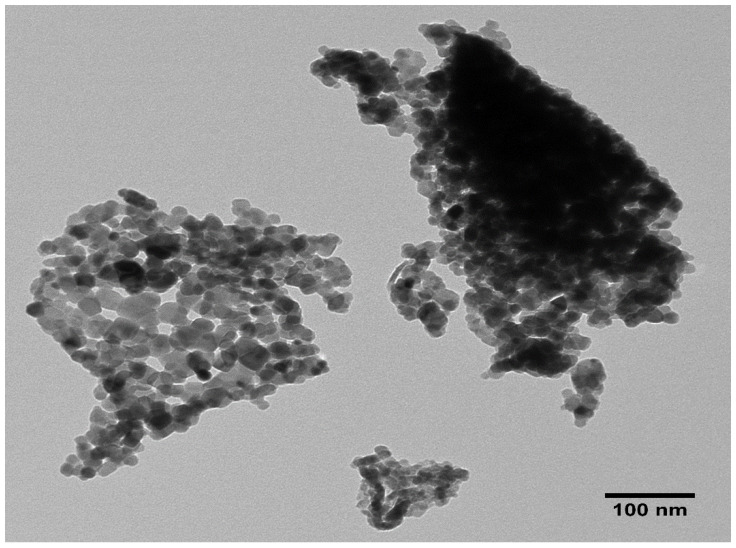
TEM micrograph of SnO_2_ NPs.

The TEM micrograph SnO_2_ NPs shown in Figure 4, exhibits two portions; one portion is formed due to the accumulation of the particles over one another forming a dark structure, whereas in the other portion the particles are somewhat evenly distributed and are closely connected with each other, leading to the formation of network structure. Although the shape of the particles are not uniform, many of the particles possess nearly spherical shape. It is also seen that the surface of the particles are smooth and have a wide range of size distribution. The particles’ size measured by ImageJ software ranges from 13.24 nm to 30.88 nm with an average size of 20.59 nm. 

### 2.5. AFM Analysis 

**Figure 5:**
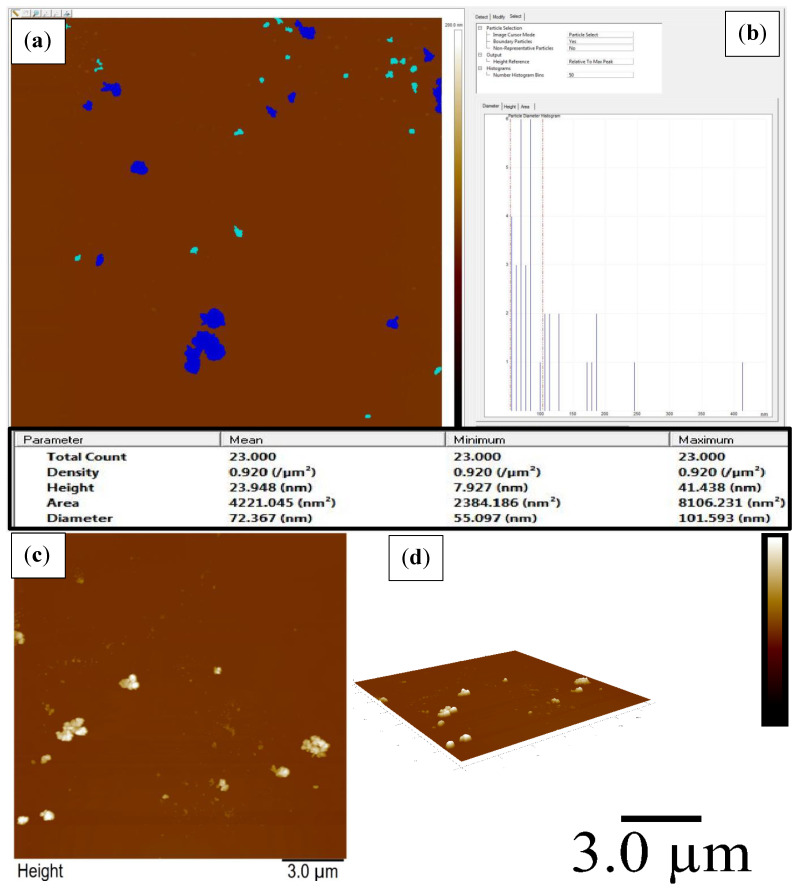
Selected area (**a**), histogram (**b**), 2-D (**c**), and 3-D (**d**) AFM micrographs of SnO_2_ NPs.

The distribution of SnO_2_ NPs of various sizes and shapes was analyzed via AFM in both 2-dimensions and 3-dimensions as shown in Figure 5. It is seen that the small particles are fused together, leading to the formation of bunch like structures. However, many tiny individual particles are also seen in the micrographs. The density of the particle is 0.920/μm^2^ whereas the height of the particles ranges from 7.93 to 41.44 nm with average height of 23.95 nm. The particles’ diameters, i.e., between 55.09 and 101.60 nm, with an average diameter of 72.73 nm.

### 2.6. DRS Analysis 

**Figure 6:**
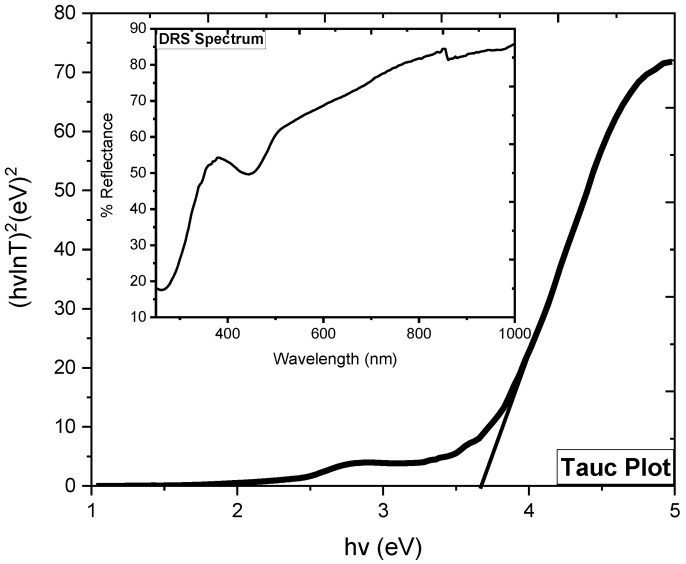
Tauc plot (inset: DRS spectrum) of SnO_2_ NPs.

The DRS spectrum of SnO_2_ NPs (inset: Figure 6) shows greater absorbance in the UV range and a clear decrease was seen in absorbance with increasing wavelength, except for a depth occurring in the boundary line UV and visible region, which might be due to some structural defects. The Tauc plot (Figure 4) was drawn to calculate the band gap energy and was noted to be 3.65 eV, almost similar to that reported in the literature [17]. 

### 2.7. FTIR Analysis 

**Figure 7:**
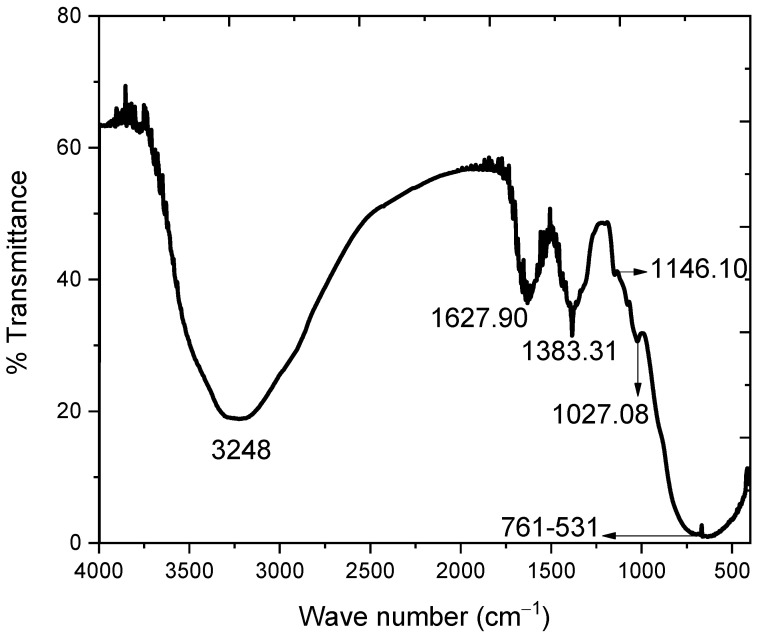
FTIR spectrum of SnO_2_ NPs.

The stretching and bending vibrations of the hydroxyl group are responsible for a broad band centered at 3248 cm^−1^ and another peak at 1627.90 cm^−1^ in the FTIR spectrum of SnO_2_ NPs (Figure 7) [18]. The signal at 1383.31 cm^−1^ confirmed the existence of NO_3_ in the sample, which might be attributable to the use of Sn(NO_3_)_2_ as a precursor in the synthesis. The peaks at 1140.44 and 1015.11 cm^−1^ are caused by Sn-OH crystal lattice vibrations [19]. The wide band in the range from 761–513 cm^−1^ is formed by the fusion of two bands at 692 and 601 cm^−1^, which are ascribed to Sn-O-Sn and Sn-O vibrations, respectively [10]. 

### 2.8. Antifungal Study

**Figure 8:**
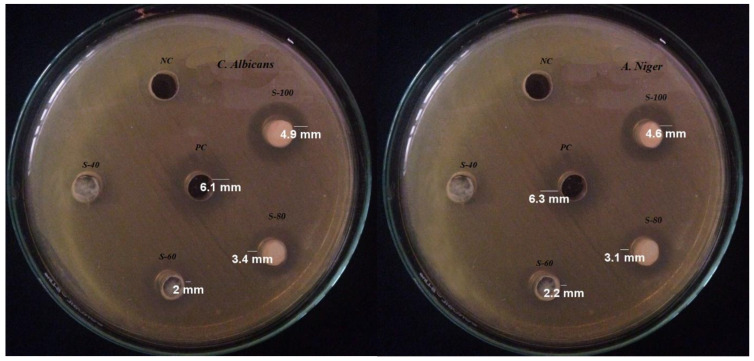
Experimental photographs of antifungal activity of SnO_2_ NPs against selected fungi at different concentrations.

The antifungal activity of SnO_2_ NPs was scrutinized against the selected fungi at different concentrations as shown in Figure 8 and the obtained data is tabulated in Table 1. The results shows that the activity of SnO_2_ NPs increased along with increase in concentration in the well. At 40 μg/mL, no activity was shown against both fungi, but onward increase in concentration significantly inhibits fungus growth and the highest activity was found at 100 μg/mL. However, the activity of SnO_2_ NPs was found to be less than the activity of the positive control, 6.1 mm and 6.3 mm for both species, respectively. The solvent was utilized as negative control and has no effect on the activity of SnO_2_ NPs and positive control. The increase in the activity with increasing concentration is attributed to the larger number of particles present in the suspension, that provide more binding sites to interact with the fungi. It has been reported that most of the antifungal agents act in a non-specific way, either changing the permeability of the cell wall and cell membrane or disturbing the cytoplasmic composition/leakage of cytoplasmic fluid. They also act as enzyme inhibitors altering the biochemical nature, which leads to the death of organisms [20]. 

### 2.9. Photocatalytic Study

**Figure 9:**
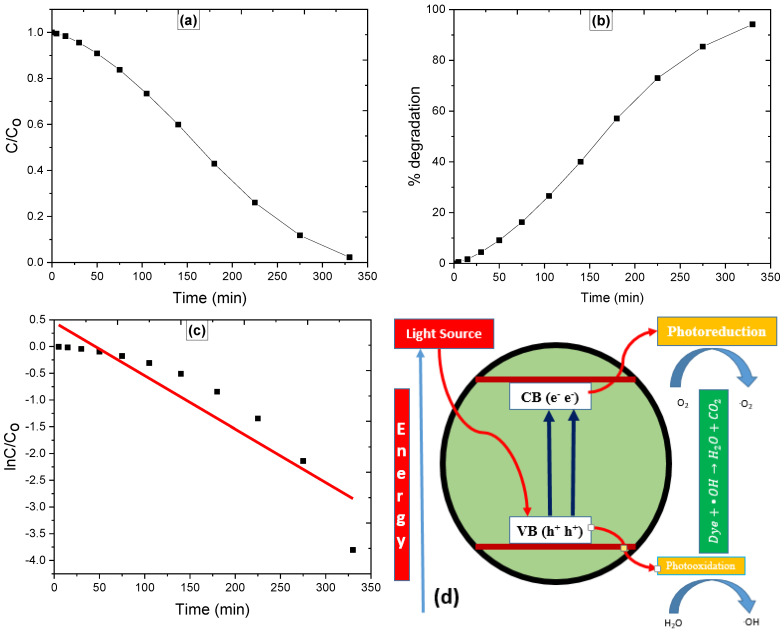
Photocatalytic parameters including, (**a**) = degradation profile, (**b**) = percentage degradation, (**c**) = kinetic plot and (**d**) = electron excitation and hole creation mechanism.

In the presence of SnO_2_ NPs, the solar light induced degradation of Rh-6G was carried out in aqueous medium, and the visual deterioration was monitored by the fading hue of the dye solution over time. The degradation process was investigated experimentally using a double beam spectrophotometer, where a decrease in the absorbance maxima at 526 nm was noted as time passed, and the results are presented in Figure 9a [21]. The percentage degradation of Rh-6G was calculated using Equation (1), and the result was 94.18 percent in 330 min (Figure 9b, which was greater than previously reported [22]. The Langmuir-Hinshelwood kinetic model (Equation (2)) was manipulated to investigate the photocatalytic reaction kinetics, where the initial and end concentrations of Rh-6G are C_o_ and C_e,_ respectively, and *k* and *t* are the apparent constants [23]. The straight line produced by plotting lnC_o_/C_e_ versus time (Figure 9c) with *r^2^* values of 0.844 suggests that the photocatalytic process is pseudo-first order. The photo-degradation rate constants for Rh-6G via SnO_2_ NPs are enumerated from the slope of linear plots and are 0.999 × 10^−2^ min^−1^.

(1) $$\%\;\mathrm{Degradation}=\frac{C_{o}-C_{t}}{C_{o}}\times 100$$

(2) ln(CCo)=−kt

When light with an energy equal to or greater than the SnO_2_ NPs band gap reaches the surface, the outermost electron is excited to the conduction band (CB), leaving a positive hole in the valence band (VB), as illustrated in Figure 9d. The positive holes interact with the water/hydroxyl group to produce hydroxyl radicals, which are powerful oxidizers that convert Rh-6G to H_2_O and CO_2_ [23]. The super oxide radicals, on the other hand, are produced by the interaction of an excited electron with absorbed oxygen, providing an additional source of hydroxyl radical and speeding up the oxidation of Rh-6G [24].

## 3. Materials and Methods

### 3.1. Materials

Sigma-Aldrich provided analytical grade chemicals such as Sn (NO_3_)_2_, hydrochloric acid and C_2_H_5_OH, which were utilized without any further purification. Deionized water was utilized to make all of the working solutions, and 15% nitric acid solution was manipulated to clean all the glassware before being bathed with the deionized water.

### 3.2. Synthesis of SnO_2_ NPs

A 10 mM solution of Sn(NO_3_)_2_ was produced on dissolving 1.21 g in 500 mL deionized water, and 80 mL from this solution was combined with 20 mL of ethanol for the fabrication of SnO_2_ NPs. The reaction mixture was stirred (250 rpm) and heated at 50 °C for 40 min at pH 2.5 by adding HCl solution. After forming a white gel and ageing it for 24 h, it was washed using deionized water and dried at 150 °C. For later usage, the white powder was kept in an airtight plastic bottle.

### 3.3. Characterization

The Panalytical X-Pert Pro X-ray diffraction model was used to investigate the crystal property, with XRD analysis in the 20°–80° 2-theta range and the Debye-Scherrer equation used to compute crystallite size. For morphological examination, a scanning electron microscope model JEOL 5910 (Japan) was utilized, and the particle size was determined using ImageJ software. At 20 keV, the energy dispersive X-ray model INCA 200 (UK) was utilized to assess the percentage composition and purity. The band gap energies were calculated for a reflectance spectrum collected using the diffuse reflectance spectroscopy model lambda 950 with a desegregating sphere in the wavelength range of 200–2500 nm. For the identification of surface functional groups, FTIR spectra in the region of 4000–400 cm^−1^ were acquired using a Nicolet 6700 (USA) spectrometer. 

### 3.4. Antifungal Assay

The antifungal screening of SnO_2_ NPs against *Aspergillus niger* (ATCC#16404) and *Candida albicans* (ATCC#10231) was carried out using the Agar well diffusion method. Four SnO_2_ NPs suspensions was prepared by ultrasonic dispersion of 40, 60, 80 and 100 μg in 1 mL. The well was bored in the media using a sterile borer and each well individually was equipped with 100 μL of each suspension and was incubated at room temperature. The zone of inhibition was computed in millimeters (mm) after 7 days as the activity of SnO_2_ NPs against the fungal species. The statistical analysis was carried out with 95% confidence interval using Microsoft Excel 2013 (Las Vegas, NV, USA). 

### 3.5. Photocatalytic Assay

The experiment was carried out in a double-walled Pyrex reactor with a water input and exit under solar light. To avoid sun contact, 50 mL of Rh-6G solution (15 ppm) and 20 mg of catalyst (0.4 g/L) were added to the reactor for each reaction, and the reactor was enclosed in aluminum foil. After exposing the reaction to sunlight for a while, 3 mL of the sample was subjected to centrifugation for 4 min at 4000 rpm and examined with a double beam spectrophotometer (Thermo Spectronic UV 500). There was a decrease in absorbance maxima as the time passed. 

## 4. Conclusions

A facile and single-step sol-gel process was operated for the fabrication of SnO_2_ NPs, which was found to be more economical and time saving, with no use of toxic and expensive templates. Different methods were used to investigate the physicochemical characteristics, which revealed the development of a well-crystalline cubic-shaped crystallite in the nano-metric range. The different shapes and morphology of SnO_2_ NPs were seen in microstructural analysis. The larger grain size might be due to the aggregation of various small particles. The EDX analysis confirmed the desired composition SnO_2_ NPs and the presence of Cl as impurity in the sample might be due to the improper washing process. A significant antifungal activity was shown by the SnO_2_ NPs against both the fungal species at higher concentrations. The 94.18 percent Rh-6G degraded in 330 min at a rate of 0.999 × 10^−2^ per min. The SnO_2_ NPs’ improved photocatalytic activity against Rh-6G was due to their tiny size and porous structure, as revealed by XRD, AFM and TEM analyses.

## Figures and Tables

**Table 1:** Antifungal activity of SnO_2_ NPs against the selected fungi and statistical analysis.

Species	Concentration (μg/mL)	Inhibition Zone (mm)	PS	NC	Variance (S2)	Standard Deviation (S)	Pearson Constant (<0.05)
*C. Albicans*	40	0	6.1	0	1.72	1.3	0.0054
	60	2					
	80	3.4					
	100	4.9					
*A. Niger*	40	0	6.3	0	1.5	1.2	0.0055
	60	2.2					
	80	3.1					
	100	4.6					

## Data Availability

All the data is enclosed in the manuscript.

## Sample Availability

Samples of the compounds are available from the authors.
